# Theoretical and experimental studies of the influence of gold ions and DMH on cyanide-free gold electrodeposition

**DOI:** 10.1039/c7ra13115a

**Published:** 2018-01-12

**Authors:** Xuefeng Ren, Maozhong An

**Affiliations:** School of Food and Environment, Dalian University of Technology Panjin 124221 China rxfhit@163.com renxuefeng@dlut.edu.cn; State Key Laboratory of Urban Water Resource and Environment, School of Chemistry and Chemical Engineering, Harbin Institute of Technology Harbin 150001 China mzan@hit.edu.cn

## Abstract

Quantum chemical calculations based on density functional theory (DFT) were employed to determine an appropriate gold source for gold electroplating and to ascertain the stable structures of gold-complexes in cyanide-free electrolyte. Based on the charge distribution of 5,5-dimethylhydantoin (DMH) and the bonding energy of gold complexes, Au^3+^ is the appropriate gold source for DMH-based gold electroplating electrolyte to get greater cathodic polarization and [Au(DMH)_4_]^−^ with 2N(4)–Au coordination structure is the most stable form of gold ion in the electrolyte. The influence of DMH, used as the complexing agent, on electrochemical behaviors was investigated using cathodic polarization, cyclic voltammetry, and chronoamperometry measurements. With DMH as the complexing agent, the cathodic polarization of gold electrodeposition was significantly enhanced. DMH concentration was determined as 0.30 mol L^−1^ based on the investigation of the influence of the DMH concentration on cathodic polarization and gold electrodeposit micromorphology. The kinetic features based on cyclic voltammogram measurements revealed that the electrodeposition was an irreversible process under diffusion control with 0.30 mol L^−1^ DMH as the complexing agent. The ion and electron transfers were obviously inhibited by DMH. The gold electrodeposition process displayed progressive nucleation according to the Scharifker and Hills nucleation model with various applied potentials. The growth rate of the crystal nucleus was reduced by DMH and promoted by a negative shift of *E*_ap_.

## Abstract 2

Au^3+^ is the appropriate gold source for the DMH-based gold electroplating electrolyte to get greater cathodic polarization.

## Graphical abstract

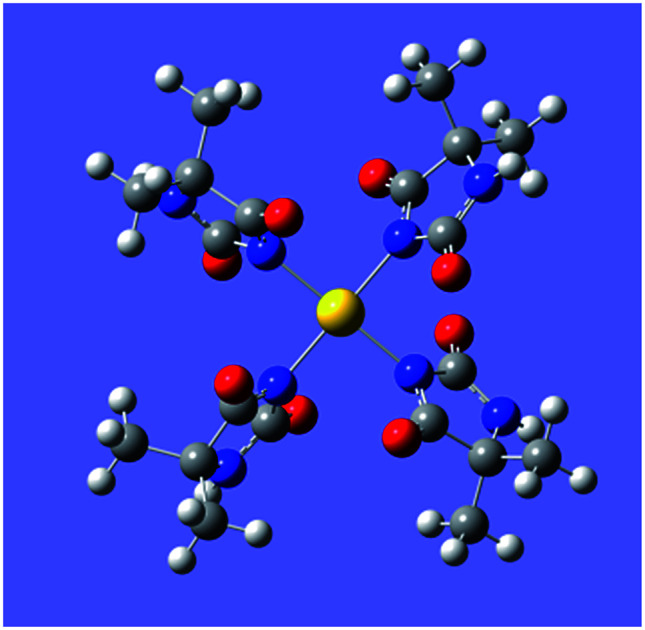

## 1. Introduction

Gold deposits are widely used for decorative and functional applications owing to their beautiful appearance and superior physicochemical properties.^1–7^ The traditional electrolytes employed for gold plating contain the cyanide-based complex [Au(CN)_2_]^−^ (ref. 8 and 9) as the source of gold, which releases free cyanide ions during the plating process. Cyanide is an extremely toxic chemical, yielding high risks to human health and the environment.^10^ Moreover, cyanide electrolytes have poor compatibility with the positive photoresists used in the microelectronic industries when the release of free cyanide occurs during the reduction of [Au(CN)_2_]^−^, which can result in photoresist cracking.^11,12^ The development of cyanide-free gold electrolytes is urgently needed and alternative complexing agents for gold plating are being investigated to avoid those problems.^13–15^ Nowadays, the main complexing agents studied and used for cyanide-free gold plating electrolytes are sulfite and thiosulfate.^16–18^ Unfortunately, these cyanide-free gold plating electrolytes still suffer from problems of instability, which limits their further study and application.^19^

A possible alternate to cyanide is DMH, a low cost and commercially available derivative of hydantoin with good solubility and stability in aqueous solutions in a large temperature range. DMH is a promising environmental friendly complexing agent, which can coordinate with many metal ions.^20–22^ The DMH-based electrolyte is simple, stable, environmentally friendly, and compatible with various chemicals and substrates. One Au^3+^ ion and four DMH^−^ ions can form a coordination complex [Au(DMH)_4_]^−^ in the electrolyte.^23^

It is important to get an insight into the bonding interaction of gold ions and DMH^−^ ions in the electrolyte as well as study the mechanisms of action of DMH on the gold electroplating process. These insights will provide important guidance to perfect the DMH-based gold electroplating electrolyte. Quantum chemical calculation and molecular modeling are fast emerging areas of research for the modeling of small chemical and biological systems in order to understand and predict their behaviors at the molecular level.^24–28^ Quantum chemical calculations based on DFT have become an effective technique for the calculation of many natural systems in pharmacology, chemistry, and biology.^29–32^ The basic foundation of DFT states that the external potential is a functional of the group-state density, and the density (an observable in 3D space) is used to describe the complicated physics behind the interactions between electrons.^33–36^ DFT has made an unparalleled impact on the application of quantum mechanics to interesting and challenging problems in chemistry for the past several decades.^37–39^ In coordinate systems, bonding interactions between metal ions and complexing agents can be predicted and investigated by quantum chemical calculations.^40–43^

In this work, the charge distributions of DMH as well as the structures of gold(i) and gold(iii) complexes were studied by quantum chemical calculations to determine the appropriate gold source for the gold electroplating. The role of DMH used as the complexing agent in cyanide-free gold electroplating electrolyte was investigated by cathodic polarization, cyclic voltammetry, and chronoamperometry. The morphology of the gold electrodeposits was evaluated by scanning electron microscope (SEM).

## 2. Experimental

### 2.1 Quantum chemical calculations

To determine the appropriate gold source for the gold electroplating and predict the possible structures of the complexing structures, we coordinated Au^+^ and Au^3+^ with DMH^−^. Quantum chemical calculations based on DFT were employed to study the charge distribution of the DMH molecule. Based on the charge distribution of the DMH molecule, the possible structures of gold(i) and gold(iii) complexes were initially identified. Then the stable structures and bonding energies of gold ions and DMH were further studied by quantum chemical calculations.

All quantum chemical calculations were carried out using the B3LYP functional method. In the geometry optimization and single point energy calculation, the 6-311++G** basis set was used for the C, N, O, and H atoms of the studied systems, except Au ions, for which we used the LANL2DZ ECP basis set. All calculations in these systems under investigation were performed using the Gaussian 09 program package at 318 K with water as the solvent in the IEFPCM theoretical model. The bonding energies *E*_bonding_ between Au^+^ or Au^3+^ and DMH^−^ were calculated using eqn (1) and (2), where *E*_Au^+^_, *E*_Au^3+^_, *E*_DMH_, *E*_H_2_O_, and *E*_H_3_O^+^_ were the energy of Au^+^, Au^3+^, DMH, H_2_O, and H_3_O^+^, respectively, and *E*_complexes_ was the energy of the gold complex.

1 *E*_bonding_ = *E*_Au^+^_ + 2*E*_DMH_ + 2*E*_H_2_O_ − *E*_complexes_ − 2*E*_H_3_O^+^_

2 *E*_bonding_ = *E*_Au^3+^_ + 4*E*_DMH_ + 4*E*_H_2_O_ − *E*_complexes_ − 4*E*_H_3_O^+^_

### 2.2 Measurements

Au^3+^ is coordinated by DMH^−^ to form the structure of [Au(DMH)_4_]^−^ as the form of main salt and the concentration of HAuCl_4_ in the electrolyte was determined as 0.025 mol L^−1^, and the various studied complexing agent DMH concentrations were 0, 0.10, 0.20, 0.30, 0.45, and 0.60 mol L^−1^. The conductive salt K_2_CO_3_ concentration was 0.36 mol L^−1^. Solutions used for Au deposition and electrochemical measurements throughout this work were prepared with analytical reagents from Sinopharm Chemical Reagent Co. Ltd using deionized water. The pH of all these electrolytes was maintained at 10.0 by KOH to guarantee the stability of the gold(iii) complexes and avoid a chemical replacement reaction occurring between the gold(iii) complexes and the metal substrate.

All electrochemical measurements were performed in a typical three-electrode cell using an electrochemical workstation at 318 K. Platinum foil with 1 cm^2^ surface area and a saturated calomel electrode (SCE) were employed as the counter electrode (CE) and the reference electrode (RE), respectively. A platinum rotating disk electrode (Pt-RDE) with a diameter of 5 mm (with 0.19625 cm^2^ surface area) was employed as the working electrode (WE) for the cathodic polarization measurements, and a glassy carbon electrode (GCE) with a diameter of 3 mm (with 0.07065 cm^2^ surface area) was used for cyclic voltammograms and chronoamperometry measurements.

Gold electrodeposits were deposited on a copper sheet coated with a nickel deposit. Gold electrodeposits were all prepared under galvanostatic conditions (8 mA cm^−2^) with mild agitation at 318 K for 5 min, either in the absence or presence of different DMH concentrations. The surface morphologies and microstructure of the gold electrodeposits were studied by field emission scanning electron microscopy (FE-SEM, Hitachi SU8000).

## 3. Results and discussion

### 3.1 Quantum chemical calculations

In the gold electrodeposition process, Au^+^ and Au^3+^ are the most commonly used main salts as gold sources; the selection of the main salt for gold electrodeposition is important for the performance of the gold deposition. Quantum chemical calculations are time- and resource-saving processes for the selection of gold ions without consumption of the extremely precious gold, the same as the investigation of Ag and Zn–Ni electrodeposition by theoretical and experimental studies.^44–46^

**Fig. 1:**
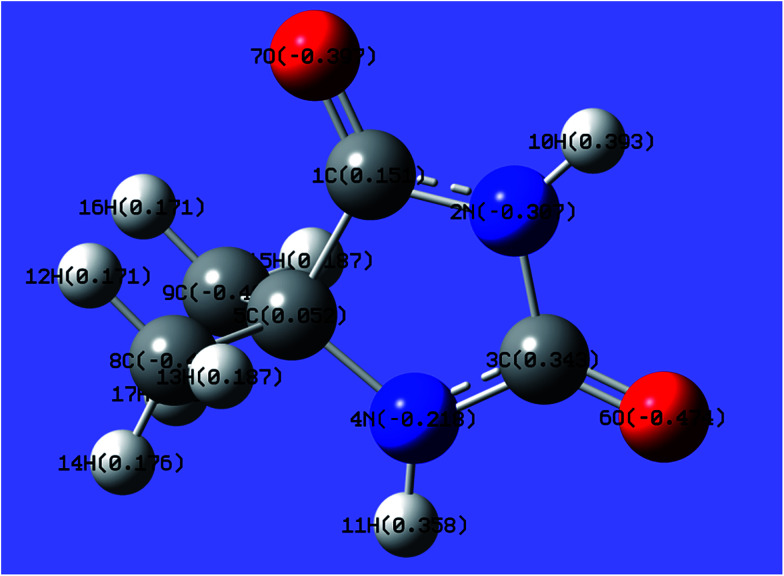
Molecular structure and charge distribution of DMH with atom symbols (unit of e).

In this work, the charge distributions of DMH as well as the structures of gold(i) and gold(iii) complexes were studied by quantum chemical calculations to determine the appropriate gold source for the gold electroplating. The molecular structure and charge distribution of DMH (a heterocyclic structure organic molecule) with atom symbols is displayed in Fig. 1.

**Fig. 2:**
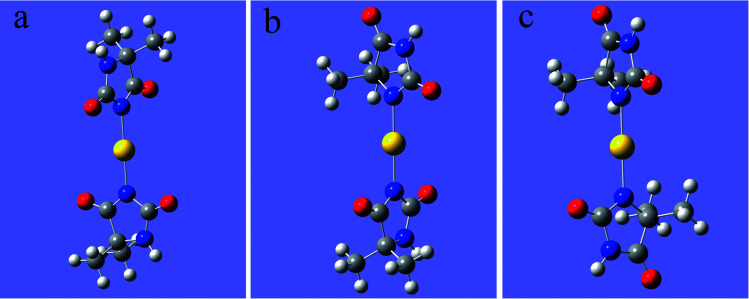
Optimized structures of the gold(i) complexes with DMH (the yellow balls represent Au; the symbols of other atoms are shown in Fig. 1), (a) 2N(2)–Au, (b) 2N(1)–Au–4N(1), (c) 4N(2)–Au.

**Fig. 3:**
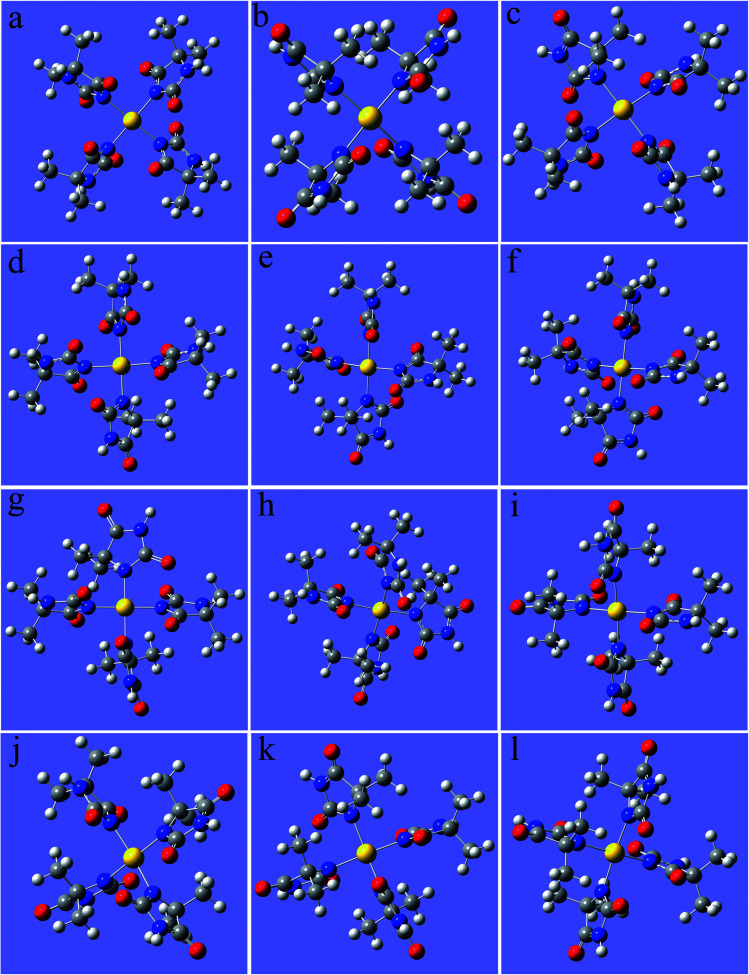
Optimized structures of the gold(iii) complexes with DMH (the yellow ball represents Au, the symbols of the other atoms are shown in Fig. 1), (a) 2N(4)–Au, (b) 4N(4)–Au, (c) 2N(3)–Au–4N(1), (d) 2N(3)–Au–4N(1), (e) 2N(3)–Au–4N(1), (f) 2N(3)–Au–4N(1), (g) 2N(2)–Au–4N(2), (h) 2N(2)–Au–4N(2), (i) 2N(1)–Au–4N(3), (j) 2N(1)–Au–4N(3), (k) 2N(1)–Au–4N(3), (l) 2N(1)–Au–4N(3).

As shown in Fig. 1, 2N (−0.307) and 4N (−0.218) of DMH were the most likely atoms to form coordinate bonds with gold ions. Three gold(i) complexes and 12 gold(iii) complexes were possible structures of gold complexes in the electrolyte according to the charge distribution of DMH. The stability of the gold(i) and gold(iii) complexes in the gold electroplating electrolyte was studied by quantum chemical calculations. The bonding energies of different complexes under study were calculated at the B3LYP/GENECP level. Fig. 2 and 3 present the optimized structures of the gold(i) and gold(iii) complexes with DMH, respectively, based on their electronic properties.

As shown in Fig. 2 and 3, these three gold(i) complexes and 12 gold(iii) complexes were stable structures in the DMH-based electrolyte. The key geometric parameters and bonding energies of these studied complexes are summarized in Tables 1–3.

**Table 1:** Energetic data calculated with B3LYP for Au^3+^, Au^+^, DMH, H_2_O, and H_3_O^+^ (units of kJ mol^−1^)

*E*_Au^3+^_	*E*_Au^+^_	*E*_DMH_	*E*_H_2_O_	*E*_H_3_O^+^_
−352 640.16	−355 088.37	−119 5927.71	−200 762.74	−201 783.32

**Table 2:** Structures and energetic data calculated with B3LYP for the gold(i) complexes (units of kJ mol^−1^)

Number	Structure	*E*_complexes_	*E*_bonding_
a	2N(2)–Au	−2 744 863.54	−39.08
b	2N(1)–Au–4N(1)	−2 744 847.35	−55.27
c	4N(2)–Au	−2 744 830.70	−71.92

**Table 3:** Structures and energetic data calculated with B3LYP for the gold(iii) complexes (units of kJ mol^−1^)

Number	Structures	*E*_complexes_	*E*_bonding_
a	2N(4)–Au	−5 133 369.61	−1100.95
b	4N(4)–Au	−5 133 267.69	−999.03
c	2N(3)–Au–4N(1)	−5 133 340.45	−1071.79
d	2N(3)–Au–4N(1)	−5 133 340.23	−1071.57
e	2N(3)–Au–4N(1)	−5 133 339.95	−1071.29
f	2N(3)–Au–4N(1)	−5 133 339.60	−1070.94
g	2N(2)–Au–4N(2)	−5 133 310.78	−1042.12
h	2N(2)–Au–4N(2)	−5 133 305.50	−1036.83
i	2N(1)–Au–4N(3)	−5 133 283.83	−1015.17
j	2N(1)–Au–4N(3)	−5 133 269.90	−1001.24
k	2N(1)–Au–4N(3)	−5 133 269.89	−1001.23
l	2N(1)–Au–4N(3)	−5 133 287.51	−1018.85

In conclusion from Tables 1–3, the large and similar bonding energies (*E*_bonding_) indicate that these three possible structures of gold(i) complexes and 12 possible structures of gold(iii) complexes were all stable structures in the gold electroplating electrolyte. With total bonding energy of −71.92 kJ mol^−1^, 4N(2)–Au of [Au(DMH)_2_]^−^ is the most stable structure of the gold(i) complexes in the DMH-based gold electroplating electrolyte. In contrast, the structure 2N(4)–Au with bonding energy of −1100.95 kJ mol^−1^ was the most possible and stable structure of gold(iii) complexes in the DMH-based gold electroplating electrolyte. The higher bonding energy of gold(iii) complexes than that of gold(i) complexes indicate that gold(iii) complexes were much more stable in the DMH-based gold electroplating electrolyte, so Au^3+^ is the appropriate gold source for the DMH-based gold electroplating electrolyte to get greater cathodic polarization. Furthermore, with the higher bonding energy, the structure 2N(4)–Au of gold(iii) complexes possessed high stability in the gold electroplating electrolyte. The conclusions of the quantum chemical calculations proved that the most stable form of gold ion was [Au(DMH)_4_]^−^ with 2N(4)–Au coordination structure in the gold electroplating electrolyte.

The results of the quantum chemical calculations confirmed that Au^3+^ is the appropriate gold source for the DMH-based gold electroplating electrolyte and [Au(DMH)_4_]^−^ with 2N(4)–Au coordination structure is the most stable form of gold(iii)-complexes in the DMH-based gold electroplating electrolyte. The influences of gold ions and DMH on the cyanide-free gold electrodeposition process were further studied to perfect the cyanide-free gold electroplating electrolyte.

### 3.2 Cathodic polarization measurement

**Fig. 4:**
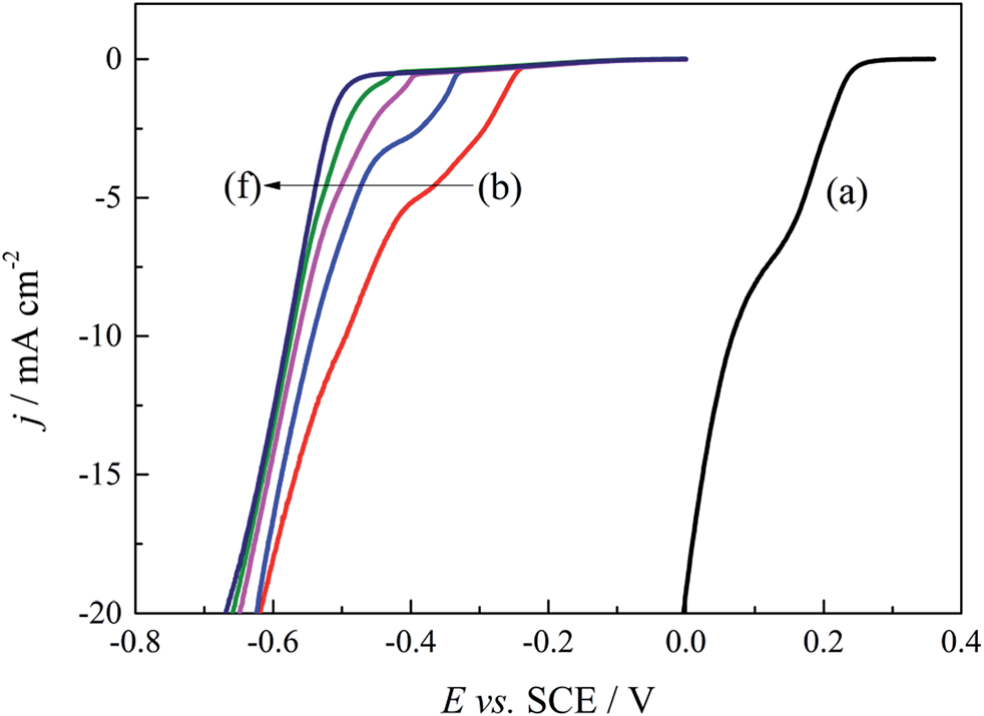
Cathodic polarization curves on the Pt-RDE at 600 rpm in the gold electroplating electrolytes with various DMH concentrations: (a) 0 mol L^−1^, (b) 0.10 mol L^−1^, (c) 0.20 mol L^−1^, (d) 0.30 mol L^−1^, (e) 0.45 mol L^−1^, and (f) 0.60 mol L^−1^.

Based on above quantum chemical calculations, Au^3+^ and DMH^−^ can form a stable 2N(4)–Au coordination complex [Au(DMH)_4_]^−^ in the cyanide-free gold electroplating electrolyte. Besides the coordination behavior, DMH can adsorb on the nickel substrate and gold surface with high stability,^47^ which can improve the cathodic polarization and the performance of the gold electrodeposit. Therefore, it is important to further optimize the concentration of DMH for excellent performance of the gold electrodeposit in this study. Cathodic polarization curves were employed to study the influences of various DMH concentrations on the gold electrodeposition process. Fig. 4 displays the cathodic polarization curves on the Pt-RDE at 600 rpm in the DMH-based gold electroplating electrolytes with various DMH concentrations. Obviously, the polarization curve measured with the electrolyte in the absence of DMH increased rapidly at 0.260 V, while the polarization curves measured in the presence of DMH all rise at potentials more negative than −0.240 V. The more than 500 mV negative shift of the onset potential demonstrates a significant kinetic difference between the solutions on the Pt-RDE without and with DMH as the complexing agent. The contrast of Fig. 4(b)–(f) revealed that the sharp increase in the potential of the cathodic polarization curves is negatively shifted with the increase of the DMH concentration, while the shift is unobvious when the DMH concentration is more than 0.30 mol L^−1^. Thus, 0.30 mol L^−1^ may be the appropriate DMH concentration applied in the investigated cyanide-free gold electroplating electrolyte.

### 3.3 Micromorphology of gold electrodeposits

**Fig. 5:**
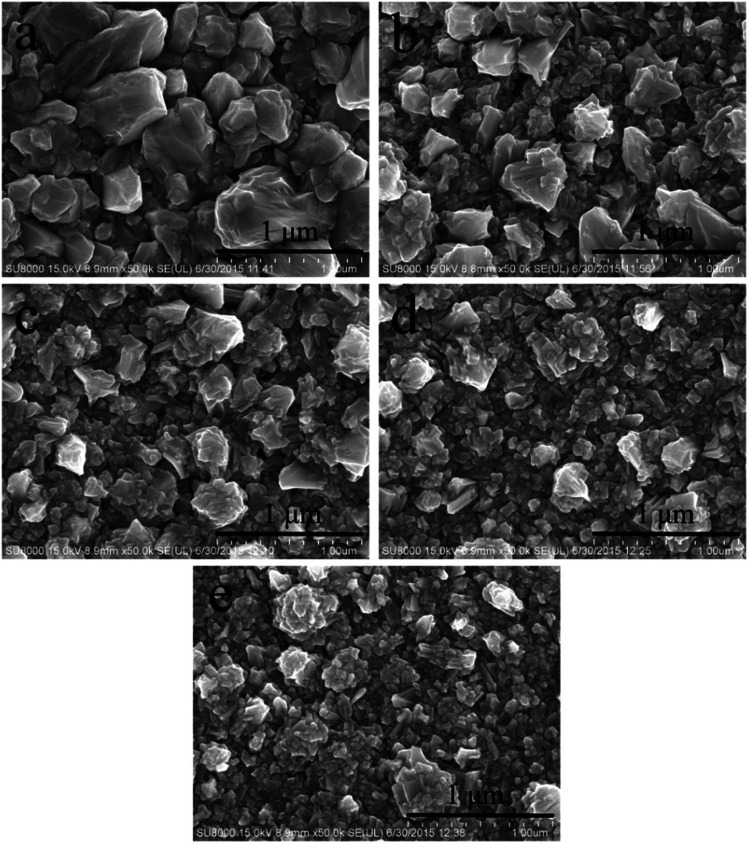
SEM images of gold electrodeposits obtained from the gold electroplating electrolytes with various DMH concentrations: (a) 0.10 mol L^−1^, (b) 0.20 mol L^−1^, (c) 0.30 mol L^−1^, (d) 0.45 mol L^−1^, and (e) 0.60 mol L^−1^.

The morphology of the gold electrodeposits obtained from cyanide-free gold electroplating electrolyte without and with DMH were further studied to evaluate the influence of DMH on the gold electrodeposition process. The appearance of the gold electrodeposits obtained from the electrolyte without DMH was reddish brown and a rough surface with severe pitting.^47^ Correspondingly, the gold electrodeposit obtained from the electrolyte with DMH was more uniform and smooth with smaller crystal particles. The SEM images shown in Fig. 5 were employed to further study the influence of the DMH concentration on the surface micromorphology of the gold electrodeposits.

The SEM images displayed in Fig. 5 confirmed that the gold electrodeposit obtained from the electrolyte in the absence of DMH was much rougher with bigger particles than that from the electrolytes in the presence of various DMH concentrations. Additionally, the performance of the gold electrodeposits can be improved with the increase of the DMH concentrations, as displayed in Fig. 5(a)–(e). The size of the crystal particles decreased with increasing DMH concentration, however, it only decreased slightly when the DMH concentration was more than 0.30 mol L^−1^. In summary, ensuring all Au^3+^ in the solution forms a stable gold(iii) complex and considering the influence of DMH on the cathodic polarization, the optimum DMH concentration was determined as 0.30 mol L^−1^ in the DMH-based cyanide-free gold electroplating electrolyte for subsequent investigations.

### 3.4 Kinetic features

**Fig. 6:**
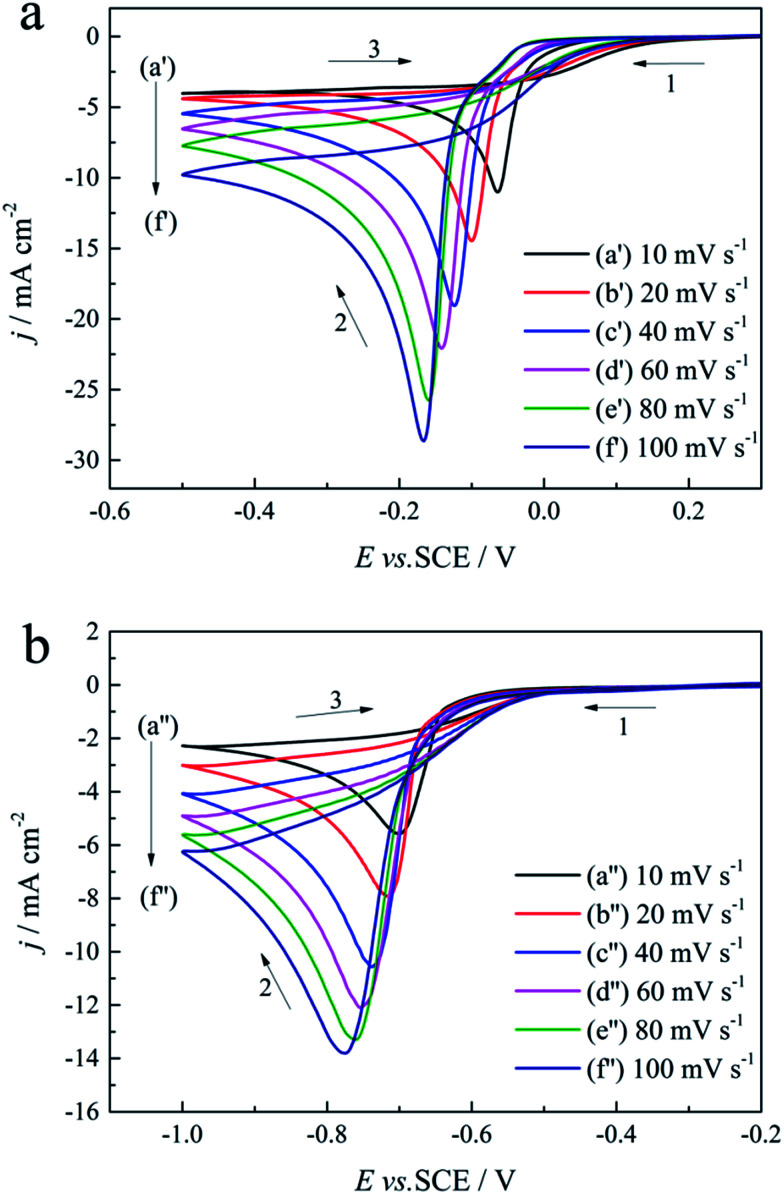
Cyclic voltammograms with different scan rates on a GCE of the gold electroplating electrolytes: (a) without DMH and (b) with 0.30 mol L^−1^ DMH.

Cyclic voltammogram measurements performed in the absence and presence of 0.30 mol L^−1^ DMH at various scan rates (10, 20, 40, 60, 80, and 100 mV s^−1^) on a GCE were employed to analyze the gold electrodeposition behavior, the control step of the gold electrodeposition, the transfer coefficient, and the diffusion coefficient influenced by DMH, as shown in Fig. 6.

As displayed in Fig. 6, the reduction peaks of the gold electroplating process in the absence and presence of DMH all shifted to more negative potentials with the increase of the scan rate, indicating that the gold electrodeposition is an irreversible process both with and without DMH as the complexing agent. At the initial scan along the negative direction, the cyclic voltammograms of the gold electrodeposition in the presence of 0.30 mol L^−1^ DMH all increase at more negative potentials than that in the absence of DMH. Furthermore, the cathodic density peaks increase with the increase of the scan rate in the electrolytes both with and without DMH. The cyclic voltammograms of the electrolytes with DMH as the complexing agent revealed a much lower cathodic density peak than that of the electrolytes in the absence of DMH at the same scan rate.

**Fig. 7:**
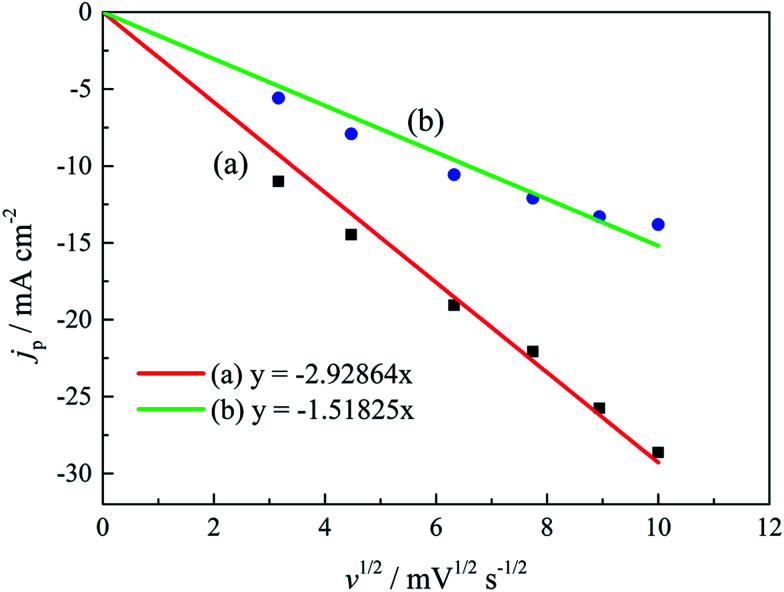
The peak current density (*j*_p_) of cyclic voltammograms with various scan rates *versus v*^1/2^ of the gold electroplating electrolytes (a) without DMH and (b) with 0.30 mol L^−1^ DMH.

The linear relationship between the peak current density (*j*_p_) and the square root of the scan rate (*v*^1/2^) is the characteristic of a diffusion-controlled reaction, which can be expressed as eqn (3):

3 *j*_p_ = 0.4958(*nF*)^3/2^(*αDv*)^1/2^(*RT*)^−1/2^*c*

where *j*_p_ is the peak current density, *n* is the number of electrons involved in the reaction (*n* = 3 in this study), *F* is the Faraday constant, *α* is the charge transfer coefficient, *D* is the diffusion coefficient, *v* is the scan rate, *R* is the gas constant, *T* is the temperature (*T* = 318 K), and *c* is the concentration of electroactive species in the electrolyte (*c* = 0.025 mol L^−1^). The fitted *j*_p_*vs. v*^1/2^ plots, presented in Fig. 7, possess a zero intercept, indicating that the electrodeposition process is diffusion controlled in the studied gold electroplating electrolyte, both with and without 0.30 mol L^−1^ DMH as the complexing agent.

The Delahay equations,^48^ as shown in eqn (4) and (5), were used to analyze the kinetic features of the gold electrodeposition process based on the cyclic voltammograms.

4 *E*_p_ − *E*_p/2_ = −1.857*RT*/(*αnF*)

**Fig. 8:**
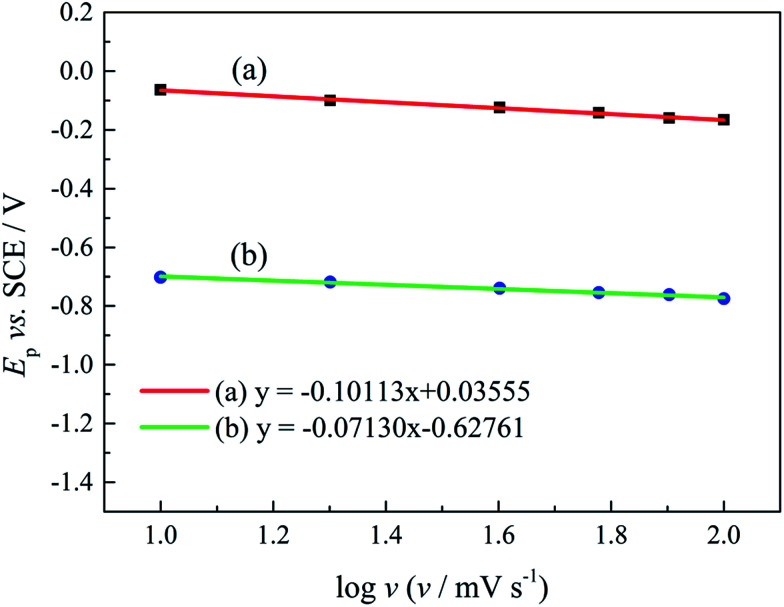
The peak potential (*E*_p_) of cyclic voltammograms with various scan rates *versus* log *v* of the gold electroplating electrolytes (a) without DMH and (b) with 0.30 mol L^−1^ DMH.

5
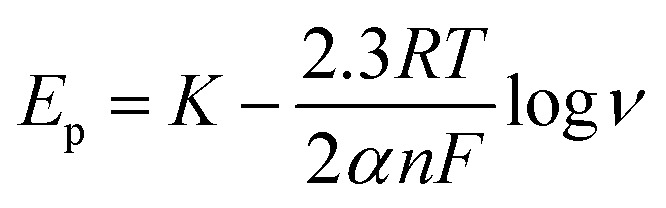
where
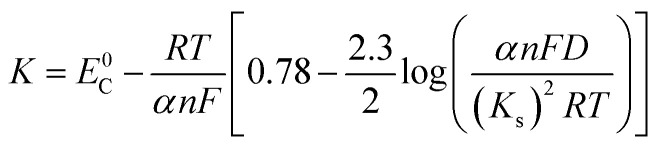
*E*_p_ is the peak potential, *E*_p/2_ is the potential at half of the peak current density*, E*^0^_C_ is the standard potential, and *K*_s_ is the kinetic constant. As presented in Fig. 8, the fitted *E*_p_*vs.* log *v* plots possess a linear feature, which proves that the gold electrodeposition in the absence or presence of DMH is a typical irreversible process under diffusion control. The kinetic features obtained from the cyclic voltammograms are listed in Table 4.

**Table 4:** The transfer coefficients (*α*) calculated from cyclic voltammetry curves at various scan rates on a GCE

*v*/mV s^−1^	DMH: 0 mol L^−1^		DMH: 0.30 mol L^−1^	
	*E*_p_ − *E*_p/2_/*V*	*α*	*E*_p_ − *E*_p/2_/*V*	*α*
10	−0.022	0.771	−0.041	0.414
20	−0.024	0.707	−0.033	0.514
40	−0.025	0.678	−0.041	0.414
60	−0.027	0.628	−0.051	0.333
80	−0.024	0.707	−0.047	0.361
100	−0.023	0.737	−0.053	0.320
Average		0.705		0.393

As displayed in Table 4, the transfer coefficients (*α*) are calculated from the cyclic voltammetry curves according to eqn (4); the *α* of the gold electroplating in the electrolyte without DMH is 0.705, bigger than that of the electrolyte with DMH as the complexing agent, which is 0.393. The almost doubling of *α* demonstrates that the electron transfer of the gold electrodeposition process was obviously influenced by the DMH in the electrolyte as the complexing agent for Au^3+^. Following eqn (3) and the fitted *j*_p_*vs. v*^1/2^ plots, the diffusion coefficient (*D*) of ions in the electrolyte were calculated as 8.69 × 10^−6^ cm^2^ s^−1^ and 4.19 × 10^−6^ cm^2^ s^−1^,^49^ respectively, indicating that *D* was reduced with the addition of DMH in the electrolyte. It indicated that with DMH as the complexing agent for Au^3+^ in the gold electroplating electrolyte, the ion transfer was inhibited. In conclusion of the kinetic features based on the cyclic voltammogram measurements, the electrodeposition processes are both irreversible under diffusion control in the studied gold electroplating electrolyte, both with and without 0.30 mol L^−1^ DMH as the complexing agent. The ion and electron transfer of the gold electrodeposition process were obviously inhibited by DMH in the electrolyte as the complexing agent for Au^3+^.

### 3.5 Nucleation mechanism

**Fig. 9:**
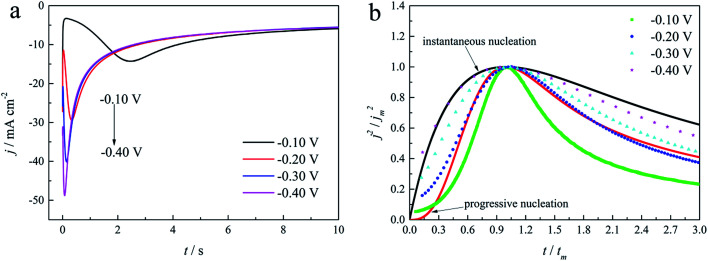
(a) Current transient curves on the GCE of electrolyte without DMH under different *E*_ap_. (b) Non-dimensional plots of instantaneous and progressive nucleation of SH models with three-dimensional nuclei growth and experimental curves of the electrolyte without DMH.

**Fig. 10:**
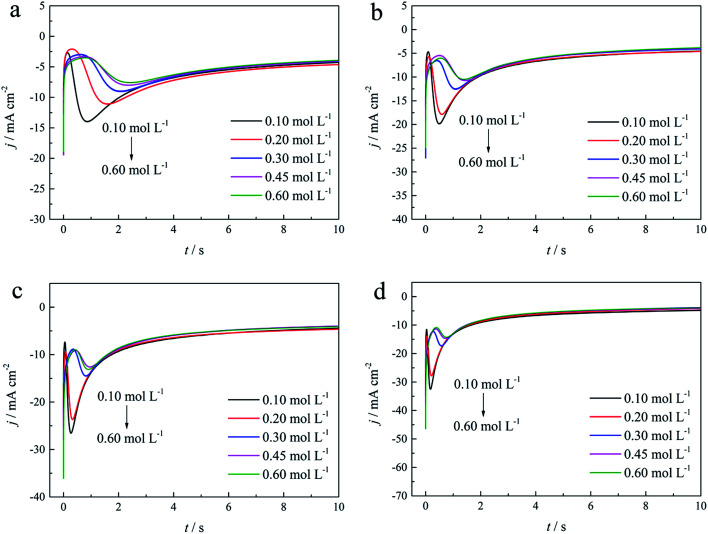
Current transient curves on the GCE of different gold electroplating electrolytes with various DMH concentrations under different *E*_ap_: (a) −0.70 V, (b) −0.75 V, (c) −0.80 V, and (d) −0.85 V.

**Fig. 11:**
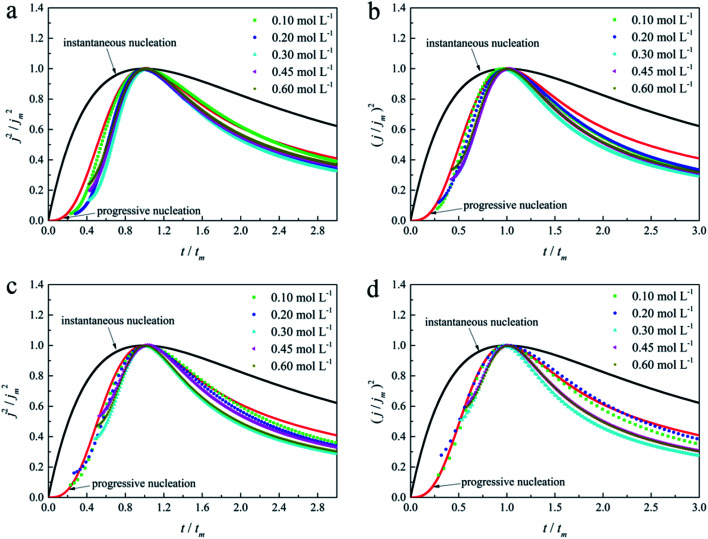
Non-dimensional plots of instantaneous and progressive nucleation of SH models with three-dimensional nuclei growth and experimental curves of the gold electroplating electrolytes with various DMH concentrations under different *E*_ap_: (a) −0.70 V, (b) −0.75 V, (c) −0.80 V, and (d) −0.85 V.

The initial nucleation mechanism and growth stage of the gold electrodeposits on the cathode during the electroplating process can be used to derive the influence of DMH on the gold electrodeposition behavior. Potentiostatic current transient curves on a GCE from electrolytes in the absence of DMH are presented in Fig. 9(a) and, comparatively, the measurements in the presence of various DMH concentrations are presented in Fig. 10. The Scharifker and Hills (SH) models for the limit cases of instantaneous and progressive nucleation were employed to analyze the current transient curves measured in this work. The SH models^50–52^ of 3-D instantaneous nucleation and progressive nucleation are shown in eqn (6) and (7), respectively:6
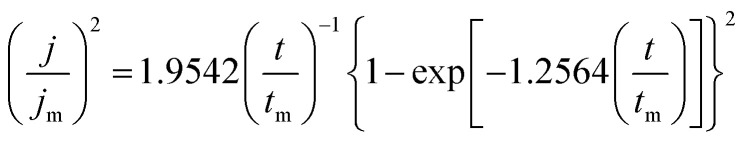
7
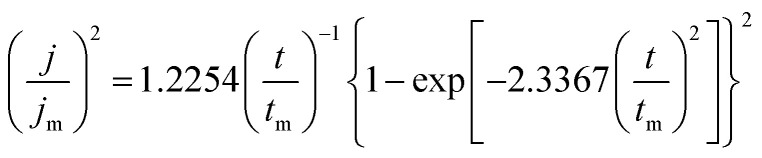
where *j* and *t* are the current density and measurement time, and *j*_m_ and *t*_m_ are the maximum current density and the corresponding time, respectively. All experimental current transient curves were transformed into non-dimensional plots of (*j*/*j*_m_)^2^*vs.* (*t*/*t*_m_) and all these plots were compared with theoretical current transient curves, as shown in Fig. 9(b) (without DMH) and Fig. 11 (with DMH).

As shown in Fig. 9(a) and 10, potentiostatic current transient curves measured in the electrolytes without DMH or with various DMH concentrations; an increase in *j*_m_ and a decrease in *t*_m_ are significantly observed when the applied potential (*E*_ap_) was set to more negative values. The existence of a current density peak in the current transient curves indicates that the charge transfer reaction controls the reaction kinetics at the early stages of the gold electrodeposition process, showing that there is a nucleation stage preceding the growth of the gold deposit. All these current transient curves can reach a steady state of current density at about 10 s of measurement, demonstrating that after the nucleation stage the reaction is under diffusion control. Moreover, as shown in Fig. 10, when the DMH concentrations increased, a decrease in *j*_m_ and an increase in *t*_m_ occurred at the same *E*_ap_, and the *j*_m_ of DMH with 0.10 and 0.20 mol L^−1^ concentrations are obviously higher than that of other concentrations.

As in the non-dimensional plots from electrolyte without DMH displayed in Fig. 9(b), when the *E*_ap_ was set to more negative values, the non-dimensional plots of experimental data transferred from following the model of progressive nucleation to instantaneous nucleation. This indicates that the nucleation model of the gold electrodeposition in the absence of DMH complexing agent is mostly influenced by *E*_ap_. In contrast with the plots from electrolytes with DMH shown in Fig. 11, the nucleation model of the gold electrodeposition process with various DMH concentrations under different *E*_ap_ is progressive nucleation. The gold nucleation process is slightly influenced by *E*_ap_ when the DMH concentrations are 0.10 and 0.20 mol L^−1^, while it is hardly influenced by *E*_ap_ when the DMH concentrations are 0.30, 0.45, and 0.60 mol L^−1^.

During the metal electrodeposition process, based on the instantaneous and progressive nucleation models, parameters of diffusion coefficient, nucleation rate constant, and nucleation density can be acquired using *j*_m_ and *t*_m_ with the equations displayed in Table 5, where *A* is the nucleation rate constant (cm^−2^ s), *N* and *N*_∞_ are the nuclei number on the electrode (cm^−2^), *n* is the number of electrons involved in the reaction (*n* = 3 in this study), *F* is the Faraday constant, *D* is the diffusion coefficient (cm^2^ s^−1^), *c* is the concentration of electroactive species in the electrolyte (*c* = 0.025 mol L^−1^), *M* is the molar mass of gold (*M* = 196.97 g mol^−1^), and *ρ* is the density of gold (*ρ* = 19.32 g cm^−3^)*.*

**Table 5:** The parameters of the SH models of progressive and instantaneous nucleation^50^

Progressive nucleation	Instantaneous nucleation
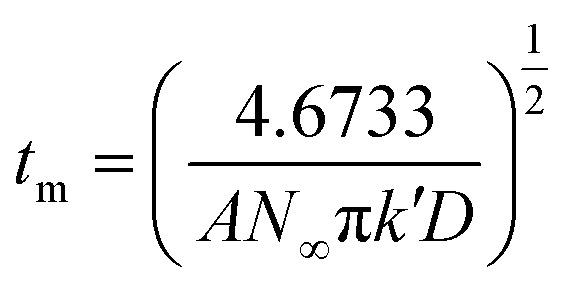	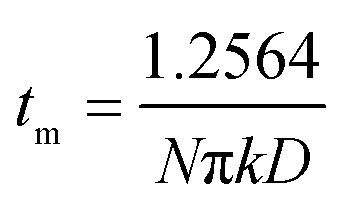
*j*_m_ = 0.4615*nFD*^3/4^*c*(*AN*_∞_*k*′)^1/4^	*j*_m_ = 0.6382π*FDc*(*Nk*)^1/2^
*j*_m_^2^ *t*_m_ = 0.2598(*nFc*)^2^*D*	*j*_m_^2^ *t*_m_ = 0.1629(*nFc*)^2^*D*
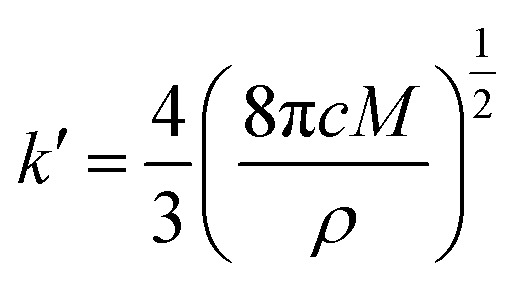	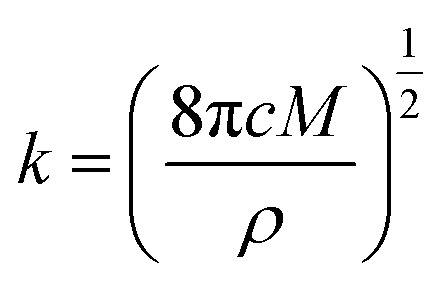

Based on the study of current transient curves, in the electrolyte without DMH, the nucleation process is mostly influenced by *E*_ap_, rather than following the instantaneous or progressive nucleation model. The parameters of the electrolyte without DMH cannot be obtained using this method. The nucleation model for the gold electrodeposition process with various DMH concentrations under different *E*_ap_ is progressive nucleation. The parameters of the nucleation rate of gold on the electrode (*AN*_∞_), the density of the saturated gold nucleation number (*N*_s_), and the epitaxial growth rate of the gold crystal (*K*_v_) can be studied with eqn (8)–(10).8
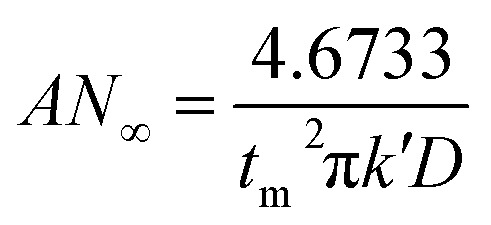
9
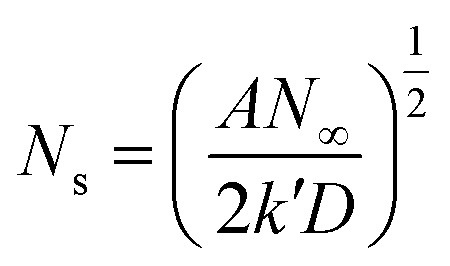
10
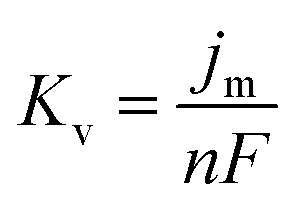


With the study of current transient curves in electrolytes with various DMH concentrations, following Table 5 and eqn (8)–(10), the *AN*_∞_, *N*_s_, and *K*_v_ can be obtained, as listed in Table 6.

**Table 6:** *AN*_∞_, *N*_s_, and *K*_v_ of gold on the GCE of the gold electroplating electrolytes with various DMH concentrations

*E* (V)	DMH (mol L^−1^)	*j*_m_ (mA cm^−2^)	*t*_m_ (s)	*AN*_∞_ (×10^7^ cm^−2^ s)	*N*_s_ (×10^7^ cm^−2^)	*K*_v_ (×10^−8^ mol s^−1^ cm^−2^)
−0.70	0.10	−13.994	0.856	4.89	2.43	4.83
−0.70	0.20	−11.111	1.614	1.16	1.08	3.84
−0.70	0.30	−9.036	2.034	0.87	1.03	3.12
−0.70	0.45	−8.020	2.350	0.72	0.98	2.77
−0.70	0.60	−7.592	2.389	0.76	1.06	2.62
−0.75	0.10	−19.84	0.509	11.56	3.41	6.86
−0.75	0.20	−17.891	0.587	9.27	3.16	6.18
−0.75	0.30	−12.579	1.106	2.80	1.80	4.35
−0.75	0.45	−10.749	1.422	1.81	1.49	3.71
−0.75	0.60	−10.494	1.395	2.01	1.62	3.63
−0.80	0.10	−26.582	0.265	45.66	7.01	9.18
−0.80	0.20	−23.694	0.323	31.73	5.94	8.19
−0.80	0.30	−14.522	0.806	5.44	2.54	5.02
−0.80	0.45	−12.600	0.930	4.70	2.53	4.35
−0.80	0.60	−13.141	0.898	4.80	2.50	4.54
−0.85	0.10	−32.512	0.176	104.17	10.63	11.23
−0.85	0.20	−27.813	0.207	87.50	10.50	9.61
−0.85	0.30	−17.325	0.581	10.20	3.43	5.98
−0.85	0.45	−14.692	0.710	7.77	3.20	5.08
−0.85	0.60	−14.282	0.742	7.21	3.10	4.93

As shown in Table 6, at the same DMH concentration, *AN*_∞_, *N*_s_, and *K*_v_ all increased with the negative shift of *E*_ap_, indicating that the growth rate of the crystal nucleus can be promoted by the negative shift of *E*_ap_. Additionally, at the same *E*_ap_, *AN*_∞_, *N*_s_, and *K*_v_ all decreased with the increase of the DMH concentration, indicating the inhibition influence of DMH on nucleation. However, when the DMH concentration reached 0.30 mol L^−1^, the downtrend of *AN*_∞_, *N*_s_, and *K*_v_ is diminished with the increase of DMH concentration, indicating that with the DMH concentration higher than 0.30 mol L^−1^, the growth rate of crystal nucleus was hardly influenced by the DMH concentration.

## 4. Conclusion

The results of the quantum chemical calculations showed that 2N (−0.307) and 4N (−0.218) of DMH were the most likely atoms to form coordinate bonds with gold ions. Three gold(i) complexes and 12 gold(iii) complexes were possible structures of gold complexes in the electrolyte according to the charge distribution of DMH. With the higher bonding energy of gold(iii) complexes than those of gold(i) complexes indicating that gold(iii) complexes were much more stable in the DMH-based gold electroplating electrolyte, Au^3+^ was the appropriate gold source for the DMH-based gold electroplating electrolyte to get greater cathodic polarization. The structure 2N(4)–Au of gold(iii) complexes possessed high stability in the gold electroplating electrolyte, which indicated that the most stable form of gold ion was [Au(DMH)_4_]^−^ with 2N(4)–Au coordination structure in the gold electroplating electrolyte.

The cathodic polarization of the gold electrodeposition was significantly enhanced with DMH as the complexing agent; the 500 mV negative shift of the onset potential demonstrates a significant kinetic change compared with the electrolyte without DMH. The sharp increase in the potential of the cathodic polarization curves and the micromorphology of the gold electrodeposits was improved with the increase of the DMH concentration. Considering the influence of the DMH concentration on the cathodic polarization and the gold electrodeposit micromorphology, the optimum DMH concentration was determined as 0.30 mol L^−1^.

In conclusion of the kinetic features based on cyclic voltammograms measurements, the electrodeposition processes are both irreversible under diffusion control in the studied gold electroplating electrolyte, both with and without 0.30 mol L^−1^ DMH as the complexing agent. The transfer coefficients of the gold electroplating electrolytes in the absence and presence of DMH were 0.705 and 0.393, respectively. The diffusion coefficients were calculated as 8.69 × 10^−6^ cm^2^ s^−1^ and 4.19 × 10^−6^ cm^2^ s^−1^ without and with DMH, respectively. The ion and electron transfer of gold electrodeposition process were obviously inhibited by DMH in the electrolyte as the complexing agent for Au^3+^. The gold electrodeposition process displayed progressive nucleation with DMH as the complexing agent according to the SH nucleation model with various applied potentials. The growth rate of the crystal nucleus was reduced by DMH and promoted by the negative shift of *E*_ap_, while when the DMH concentration was higher than 0.30 mol L^−1^, the growth rate of the crystal nucleus was hardly influenced by the DMH concentration.

## Conflicts of interest

There are no conflicts to declare.

## Supplementary Material
